# The relationship between dietary patterns and aggressive behavior in adolescent girls: A cross‐sectional study

**DOI:** 10.1002/brb3.2782

**Published:** 2022-10-28

**Authors:** Mahsa Malekahmadi, Sayyed Saeid Khayyatzadeh, Javad Heshmati, Shadia Hamoud Alshahrani, Nikzad Oraee, Gordon A. Ferns, Safieh Firouzi, Naseh Pahlavani, Majid Ghayour‐Mobarhan

**Affiliations:** ^1^ Anesthesiology and Intensive Care Department, Imam Khomeini Hospital Complex Tehran University of Medical Sciences Tehran Iran; ^2^ Department of Clinical Nutrition School of Nutritional Sciences and Dietetics Tehran University of Medical Sciences Tehran Iran; ^3^ Nutrition and Food Security Research Center Shahid Sadoughi University of Medical Sciences Yazd Iran; ^4^ Songhor Healthcare Center Kermanshah University of Medical Sciences Kermanshah Iran; ^5^ Medical Surgical Nursing Department King Khalid University Khamis Mushate Saudi Arabia; ^6^ Centre for Environmental Policy Imperial College London London UK; ^7^ Brighton & Sussex Medical School Division of Medical Education, Falmer Brighton Sussex UK; ^8^ Department of Nutritional Sciences Faculty of Health Golestan University of Medical Sciences Gorgan Iran; ^9^ Health Sciences Research Center Torbat Heydariyeh University of Medical Sciences Torbat Heydariyeh Iran; ^10^ Metabolic Syndrome Research Center Mashhad University of Medical Sciences Mashhad Iran

**Keywords:** adolescent, aggression, dietary patterns, factor analyses

## Abstract

**Background:**

There are few studies about the relationship between dietary patterns and aggression. The aim of this study was to assess the relationship between the main dietary patterns and aggression scores among adolescent girls in Iran.

**Methods:**

This cross‐sectional study was conducted on 670 adolescent girls. The 168‐item self‐administered Semi‐quantitative Food Frequency Questionnaire was used to evaluate dietary intake and to identify major dietary patterns, while factor analysis was applied. Aggression was evaluated by the validated Persian version of the Buss‐Perry questionnaire. Statistical analysis was performed by crude and adjusted models.

**Results:**

Three main dietary patterns including healthy, fast food, and Western were identified. A significant positive association was found between more adherence to Western dietary pattern and the presence of a high aggression score (OR: 2.00; 95% CI: 1.32–3.05, *p*‐_trend =_ .001); even after adjustment for potential confounders, these findings were significant.

**Conclusion:**

Although Western dietary patterns were associated with increased aggression risk, there was no significant relationship between healthy and fast food dietary patterns and the prevalence of a high aggression score. Further studies, particularly longitudinal intervention studies, are required to clarify this relationship.

## 1 INTRODUCTION

In the previous years, the prevalence of psychological disorders has increased, and studies have shown that dietary patterns can affect the consequences of these disorders (Hosseinzadeh et al., 2016; Mousavi et al., 2022). Aggression is known as one of these psychological disorders that in psychology is defined as a tendency to violent behavior that can cause physical or psychological noxiousness to human (Anderson & Bushman, 2002). Aggression causes a threat to social safety and potentially leads to severe criminality, risking the safety of potential victims and causing harm, and imposing costs to society as a whole (Dambacher et al., 2015). Physical and verbal aggression is common in adolescent females (Cotter & Smokowski, 2016; Fagan et al., 2017). The prevalence of behavioral disorders in female adolescents suggests that there is a likelihood of comorbidities with aggressive behavior (Merikangas et al., 2010). The prevalence of aggression among the Iranian adolescents of both sexes has been estimated to be 30% to 65.5% (Sadeghi et al., 2014), while the differences between the rate of behavioral disorders between male and female subjects are marginally significant (Lahey et al., 2006; Odgers & Moretti, 2002). However, female aggression has been reported to have increased by 86%, while male aggression has increased by 17% in the past twenty years (Puzzanchera & Hockenberry, 2012). Thus, female aggression has had a significantly greater increase compared to male aggression, and yet most of the adolescent aggression studies have focused on men (Berkout et al., 2011; Kerig, 2014; Valois et al., 2002).

Various lifestyle factors, such as watching television, stress, socioeconomic variables, and dietary factors, are related to adolescent aggression (Laninga‐Wijnen et al., 2016; Pahlavani et al., 2014; Taubner et al., 2016). There is evidence to suggest that diet can affect health‐related indicators, albeit not significantly (Khayyatzadeh et al., 2018; Pahlavani et al., 2020). Austin et al. (2014) found that supplementation of multivitamins and proteins in military personnel could alleviate aggressive behavior. While McNamara and Strawn (2013) found that long‐chain omega 3 fatty acid may affect the state of anger when inflammatory cytokines levels are raised, such that supplementation of this nutritious factor could help to minimize nervousness (Rasad et al., 2014). Furthermore, Zahedi et al. (2014) found that the intake of “*junk foods*” (high energy and low nutritional content foods) in adolescents and children may increase the risk for psychiatric disorders and aggressive behaviors. Another study has indicated that high consumption of dairy products reduced state‐trait anger expression in female students (Kalantari et al., 2016).

No studies have investigated the relationship between habitual dietary patterns and occurrence of aggression among female adolescents in Iran, even though such a study could be widely applied in interdisciplinary fields. This study can be extended to different regions of the world, as different cultures and geographical areas coupled with religious beliefs affect dietary patterns, which could lead to different trends in aggression in the same subject group (Hu, 2002; Kant et al., 2004; Mansouri et al., 2020). As such, the paper aims to explore this area of research and provide food for thought for further studies.

## 2 METHODS

### 2.1 Study population

The present study was a cross‐sectional study that started in January 2015, 854 individuals were invited to participate in the study initially, and finally 670 adolescent girls were selected and recruited using a random cluster sampling method from several schools in different areas of Razavi Khorasan Province to participate in the study. Inclusion criteria were the age of 12–18 years with no history of any chronic diseases (colitis, diabetes, cardiovascular diseases, cancer, and hepatitis). Also, we initially excluded all participants who were suffering from chronic diseases such as hypertension, diabetes, and so forth and were taking related medications, as well as subjects who were taking supplements such as vitamin D, calcium, and so on were not included in the study. This information was obtained using a checklist designed by the research team.

At first, all of the subjects filled written informed consent forms; the study was approved by The Ethics Committee of Mashhad University of Medical Sciences.

### 2.2 Assessment of demographic status

Trained interviewers collected the general characteristics of subjects including age, smoking status, menstruation status, and medical history. Physical activity was assessed by the validated Modifiable Activity Questionnaire (MAQ) (Delshad et al., 2015), and the level of physical activity was estimated based on metabolic equivalent task (MET) minutes per week.

### 2.3 Dietary assessment

Dietary intakes of participants were collected by a valid and reliable food frequency questionnaire (FFQ) containing 168 food items (Esfahani et al., 2010). Subjects had nine multiple choice frequency response categories for each food, ranging from “never or < 1/month” to “12/day.” Since the Iranian culture and food habits were judged similar to that of Americans, the US Department of Agriculture's (USDA) national nutrient databank was used to estimate the daily nutrient intake (Esfahani et al., 2010). Finally, 40 food groups were defined to extract major dietary patterns (see Table S1).

### 2.4 Anthropometric and biochemical assessment

Anthropometric measurements including weight, height, and waist circumferences (WC) were estimated by using standard protocols. All anthropometric measurements were done twice, and the mean values were reported, respectively. Blood pressure was measured using a standardized protocol, and averages were recorded.

### 2.5 Assessment of aggression

Aggression score was assessed using a validated Persian translation of the Buss‐Perry questionnaire (Zivari‐Rahman et al., 2012). This questionnaire includes 29 questions with multiple choice responses.

Median cut‐point for the definition of aggression was used; such that subjects were categorized as aggressive if their score was below 64.

### 2.6 Statistical methods

Three major dietary patterns based on 40 food groups were identified by a principal component analysis, and factors were rotated using the varimax rotation technique. Furthermore, three factors were determined in relation to Eigen values > 1.5 and the interpretation of scree plot. Therefore, according to our data interpretation and the previous studies, three major dietary patterns were defined by considering factor loading and total intakes of weighted foods; then, the scores of each pattern were calculated for each subject factor (Khani et al., 2004; Oddy et al., 2009). Individuals were classified according to tertiles of dietary pattern scores. A one‐way analysis of variance was performed in order to assess the differences in continuous variables across tertiles in categories of three dietary pattern scores; moreover, chi‐squared test was used for assessing the categories across tertiles of dietary pattern in participants. To investigate the relationship between dietary patterns and aggression presence, logistic regression was used in several models. In model I, age and energy intake were controlled. In model II, the same factors were assessed together with physical activity and menstruation. Body mass index (BMI) was added as a factor in model III. Total trends of the odds ratios through ascending tertiles of dietary patterns scores were assessed using the Mantel‐Haenszel extension. A *P*‐value < .05 was considered statistically significant. For all statistical analyses, SPSS software version 15.0 (SPSS Inc., Chicago, IL, USA) was used.

## 3 RESULTS

### 3.1 Identification of major dietary patterns

The following three major dietary patterns were identified: (i) the “first” dietary pattern (healthy), which was high in vegetables, eggs, yoghurt, cruciferous vegetables, tomatoes, green leafy vegetables, garlic, fruits, low and high fat dietary products; (ii) the “second” dietary pattern (fast food) which was greatly laden with potato, hydrogenated fats, sugars, tea, salt, and spices; (iii) the “third” dietary pattern (Western) which was rich in snacks, red meat, poultry, industrial fruit juices and compote, soft drinks, sweets, and desserts. Overall, these dietary patterns accounted for 18.38% of the variance in dietary intakes (see Table S2).

### 3.2 General characteristics and dietary intakes of study participants

General characteristics of study participants across tertiles of dietary patterns are presented in Table 1. Among general characteristics, physical activity for first pattern in the third tertile was significantly higher than that of the first tertile. According to the anthropometric measurements, the BMI for participants, who were in tertile 1 of second dietary pattern, was significantly higher than that of participants who were in tertile 3. No significant differences in the distribution of other variables in terms of tertiles of dietary patterns were observed.

**TABLE 1:** Demographic and anthropometric measurements of study participants by tertiles (Q) categories of dietary pattern score

	Healthy pattern			Fast food pattern			Western pattern		
Tertile	Tertile 1	Tertile 3	*P*‐value^a^	Tertile 1	Tertile 3	*P*‐value	Tertile 1	Tertile 3	*P*‐value
Age (y)^b^	14.5 ± 1.5	14.5 ± 1.5	.9	14.4 ± 1.6	14.6 ± 1.5	.24	14.4 ± 1.4	14.4 ± 1.5	.19
Weight (kg)	51.6 ± 12.4	53.7 ± 11.9	.19	53.9 ± 13.2	52.8 ± 13.4	.1	53.6 ± 14.7	52.9 ± 11.5	.21
BMI (kg/m^2^)	20.6 ± 3.7	21.6 ± 5.04	.05	21.7 ± 4.6	21.2 ± 4.8	.04	21.5 ± 5.4	21.2 ± 4.05	.3
Waist circumference (cm)	69.8 ± 8.1	70.9 ± 9.1	.41	70.8 ± 9.5	70 ± 9.3	.45	70.7 ± 9.6	70.5 ± 8.4	.25
Physical activity (MET)	44.8 ± 2.8	45.8 ± 3.9	.006	45.1 ± 3.3	45.3 ± 3.5	.64	44.9 ± 2.7	45.5 ± 3.8	.1
Systolic blood pressure (mmHg)	96.8 ± 13.3	97.4 ± 13.9	.32	96.4 ± 13.9	95.6 ± 13.1	.31	96.9 ± 14.1	95.9 ± 15.3	.67
Diastolic blood pressure (mmHg)	62.8 ± 12.2	62.8 ± 13.3	.99	62.6 ± 13.5	62.5 ± 13.3	.76	61.9 ± 13.3	62.8 ± 13.6	.31
Passive smoker (%) (yes)	35.6	27.8	.18	29.3	30.2	.33	32	31.4	.98
Menstruation (%) (yes)	87	87.8	.91	86.7	85.8	.2	85.5	87.5	.28

^a^ ANOVA for continuous variables and chi‐squared test for categorical variables.

^b^ All values are mean ± SD.

Subjects in the third tertile of first pattern had a higher intake of energy, low and high fat dairy, fruits, vegetables, legumes, but lower intake of sugar, soft drink, and snacks compared with those in the first tertile. The participants in the highest tertile of the second pattern had higher intakes of energy, spices, hydrogenated fat, sugar, soft drink, snack, and sweet and dessert but lower intake of high fat dairy and red meat compared with those in the lowest tertile.

Individuals in the third tertile of third pattern had higher intakes of energy, red meat, processed meat, low and high fat dairy, soft drinks, snack and sweet and desserts but lower intake of legumes, spices, hydrogenated fat, and sugar compared with those in the first tertile (Table 2).

**TABLE 2:** Dietary intakes of study participants by tertiles (Q) categories of dietary pattern scores

	Healthy pattern			Fast food pattern			Western pattern		
	Tertile 1	Tertile 3	*P*‐value^a^	Tertile 1	Tertile 3	*P*‐value	Tertile 1	Tertile 3	*P*‐value
**Food groups (g/1000 Kcal)**									
Energy (Kcal/d)	2414.8 ± 846.1	3101.1 ± 731.1	<.001	2359.9 ± 830.6	3049.5 ± 770.4	<.001	2237.6 ± 776.6	3301.2 ± 662.7	<.001
Red meat^b^	4.77 ± 5	5.44 ± 5.81	.2	5.59 ± 4.69	4.24 ± 3.99	.001	3.66 ± 3.5	6.47 ± 6.39	<.001
Processed meat	1.81 ± 2.38	1.79 ± 2.68	.92	1.97 ± 2.96	1.58 ± 1.94	.15	0.94 ± 1.24	2.84 ± 3.39	<.001
Low fat dairy	54.1 ± 52.07	99.4 ± 73.3	<.001	85.8 ± 69.7	73.2 ± 67.3	.09	92.3 ± 75.5	69.2 ± 55.3	.001
High fat dairy	45.1 ± 44.3	79.1 ± 67.5	<.001	73.5 ± 65.6	51.7 ± 42.1	<.001	56 ± 52.5	61.6 ± 48.7	.01
Fruit	62.02 ± 56.2	97.4 ± 68.2	<.001	82.6 ± 70	73.8 ± 61.8	.28	71.5 ± 51.3	83.1 ± 71.6	.1
Vegetable	50.5 ± 29.5	130.6 ± 73.2	<.001	89.4 ± 61.2	90.4 ± 68.5	.59	101.4 ± 69.6	77.2 ± 53.6	<.001
Legumes	30.8 ± 26.5	34.4 ± 26.6	.05	31.8 ± 25.2	30.1 ± 22.3	.51	34.6 ± 27.1	28.4 ± 20.2	.01
Spices	1.05 ± 1.11	1.03 ± 1.11	.89	0.63 ± 0.66	1.51 ± 1.51	<.001	1.2 ± 1.4	0.92 ± 0.89	.01
Hydrogenated fat	8.03 ± 10.1	7.7 ± 8.9	.46	4.3 ± 5.6	11.3 ± 11.2	<.001	10.4 ± 11.6	5.35 ± 6.08	<.001
Sugar	5.02 ± 5.91	3.44 ± 3.25	.001	1.6 ± 1.95	6.8 ± 6.1	<.001	4.71 ± 5.52	3.51 ± 3.53	.01
Soft drink	27.7 ± 48.01	15.7 ± 29.1	<.001	12.9 ± 30.2	28.5 ± 45.4	<.001	8.51 ± 13.07	35.6 ± 52.03	<.001
Snack	12.8 ± 13.4	8.6 ± 8.4	<.001	8.9 ± 9.8	12.3 ± 11.1	.001	6.25 ± 6.6	15.3 ± 12.5	<.001
Sweet and dessert	14.5 ± 11.5	12.6 ± 10.9	.1	11.9 ± 10	15.3 ± 12.3	.002	9.88 ± 8.63	17.2 ± 12.04	<.001

^a^ Obtained from one‐way ANOVA.

^b^ All values are mean ± SD.

### 3.3 The relationship between three habitual dietary patterns and aggression

Multivariable‐adjusted odds ratios for aggression scores across the tertile categories of dietary pattern are shown in Table 3. No significant associations were found between the first [*p_trend_* = .57, odds ratio and confidence interval for second and third tertile were, respectively: OR:1.44, 95% CI: 0.95,2.19, OR: 0.89, 95% CI: 0.6,1.32] and second dietary patterns [*p_tre_*_nd_ = .39, odds ratio and confidence interval for second and third tertile were respectively: OR: 0.73, 95% CI: 0.49,1.09, OR: 1.2, 95% CI: 0.79,1.82] and aggression score following the application of multivariate models. The participants in the highest tertile of third dietary pattern had a significantly greater odds ratio for aggression (with and without adjusting for variables in Table 3) [*p_trend_* for crude model = .001, odds ratio and confidence interval for tertile 2 and 3 were, respectively: OR: 1.18, 95% CI: 0.8,1.74, OR: 2.00, 95% CI: 1.32–3.05 and *p_trend_* After adjustment for potential confounding variables was .0006, and odds ratio and confidence interval for tertile 2 and 3 were, respectively: OR: 1.17, 95% CI: 0.78,1.76, OR: 2.04, 95% CI: 1.24,3.35].

**TABLE 3:** Multivariate adjusted odds ratios (95% CIs) for aggression (Q) categories of dietary pattern scores

	Crude	Model I^a^	Model II^b^	Model III^c^
Healthy pattern				
Tertile 1	1	1	1	1
Tertile 2	1.44 (0.95–2.19)	1.4 (0.92–2.14)	1.43 (0.93–2.19)	1.47 (0.96–2.27)
Tertile 3	0.89 (0.6–1.32)	0.77 (0.51–1.19)	0.78 (0.51–1.21)	0.8 (0.51–1.23)
*P* trend^d^	.57	.88	.31	.34
Fast food pattern				
Tertile 1	1	1	1	1
Tertile 2	0.73 (0.49–1.09)	0.66 (0.43–0.99)	0.64 (0.42–0.98)	0.62 (0.41–0.96)
Tertile 3	1.2 (0.79–1.82)	1.01 (0.65–1.58)	0.94 (0.59–1.49)	0.92 (0.58–1.46)
*P* trend^d^	.39	.95	.8	.73
Western pattern				
Tertile 1	1	1	1	1
Tertile 2	1.18 (0.8–1.74)	1.18 (0.8–1.76)	1.2(0.8–1.81)	1.17 (0.78–1.76)
Tertile 3	2.00 (1.32–3.05)	2.00 (1.23–3.25)	2.02 (1.23–3.32)	2.04 (1.24–3.35)
*P* trend^d^	.001	.006	.006	.006

^a^ Adjusted for age and energy intake.

^b^ Additionally adjusted for passive smoking, Physical activity, menstruation, drug and supplement use.

^c^ Additionally adjusted for BMI.

^d^ Resulted from Mantel‐Haenszel extension c2 test.

## 4 DISCUSSION

Three main dietary patterns in the population of female adolescents were detected. The individuals in the third tertile of Western dietary pattern had a higher prevalence of a high aggression score. No other significant associations were observed between the healthy or fast food dietary patterns and aggression. To the authors’ best knowledge, this study is the first to link the main habitual dietary patterns and aggression.

Aggression is a common behavioral problem among adolescents, which is characterized by offensive verbal and physical behaviors (Cotter & Smokowski, 2016). Diet is among the factors that play an important role in the control of aggression (Han & Dingemanse, 2017). This paper demonstrates that habitual dietary patterns may contribute to the alleviation or worsening of aggression. Therefore, the improvement of diet quality and the avoidance unhealthy diets may lead to the reduction of aggressiveness in adolescents, although this will need to be confirmed by longitudinal intervention studies. Adolescence is the most sensitive period in terms of emotions and body perception, where teenagers are under great influence from their peers; this peer influence has been seen to adversely affect food choices by way of, for example, weight loss or other unsound beliefs, which may lead to a lower intake of essential nutrients (Berkout et al., 2011; Cotter & Smokowski, 2016; Kalantari et al., 2016).

The role of diet and nutrients in antisocial behavior has been considered in clinical trials (Benton, 2007). Meta‐analysis of five double‐blind placebo‐controlled trials revealed that children were potentially influenced by a wide range of food items, but the pattern was proprietary to the child (Benton, 2007). However, it is clear that food nutrient policies in schools, where children and adolescents spend 30 h a week and consume over a third of their meals, has had an overall positive impact on students’ food choices (Van Ansem et al., 2013). Therefore, it can be asserted with some degree of certainty that public health policies have been successful in forming the eating habits of students.

Leading from the earlier parts of the current paper on the effect of dietary habits on aggression, it can be asserted by extension, therefore, that increasing food security and availability in schools and the local areas will have beneficial effects on reducing aggression rates among female adolescents (Helton et al., 2019; World Health Organization, 2018). There is no evidence to suggest that the results from the implementation of nutrient food policies in Iran will differ from the policies examined above. Therefore, as a matter of public policy, it is suggested that local and provincial policy makers in Iran also begin to adopt best practices from the vast array of school nutrient food policies in order to tackle public health problems such as aggression in female adolescents.

The current study showed that individuals who were in the third tertile, so‐called Western diet pattern, which was rich in red and processed meat (high protein), experienced high aggression scores. Other studies focused on behavioral changes as a consequence of nutrient status. A significant and strong association between soft drinks consumption, and violence was observed in the study of Boston public high schools, where the participants were asked how often they drank nondiet soft drinks and engaged in physical violence with a peer. A direct cause‐and‐effect relationship between sugary drinks consumption and violence is not untenable in such circumstance; such that the sugar or caffeine content of soft drinks is sometimes found to be an intervening factor in reducing one's self esteem which may result in aggression (other factors could also have played a role which may have been unaccounted for in this analyses) (Solnick & Hemenway, 2012); their findings were in line with those of the current study. When ecological and individual factors were controlled in Moore's study, children at the age of 10 who ate confectioneries on a daily basis were considerably more likely to depict violent behaviors at age 34 years (Moore et al., 2009). That may be due to high concentration of simple sugar, saturated, and transfatty acids that alter the mental functions. Conversely, a healthy lifestyle was linked with a relatively lower association of being frequently violent in developing countries (Turagabeci et al., 2008). However, the current study did not find a significant effect of a healthy dietary pattern as a component of healthy lifestyle on violence in female adolescents. Nevertheless, studies have shown results similar to those in the current paper, such that the intake of high energy and low nutritional content foods may increase the risk for psychiatric distress and violent behaviors in children and adolescents, aged 6 to 18 years old in Iran (Zahedi et al., 2014). Another study indicated a higher consumption of dairy products reduced state‐trait anger expression in female students (Kalantari et al., 2016), whereas in this papers, in the third tertile of third dietary pattern that intake of dairy products was high, the risk of aggression was also high. This anomaly may be due to the effect of another component of third dietary pattern, for example, the effect of red and processed meat was dominant.

In their study, Feingold assumed that salicylates (chemicals in food with a similar structure to aspirin), which is a chemical component used as an additive to processed foods like processed meat, cause hyperactivity (Benton, 2007). According to this theory, the reason that the third tertile of Western pattern were related to more aggression will be explained.

Psychological disorders, including aggression, are the result of interactions between genetics, the body's immune status, hormones, and biochemical indices (Hillbrand & Spitz, 1999; Maes, 1999). However, the mechanisms of this is complex and not completely understood. Therefore, diet and food patterns can affect brain and psychological function by playing a role in mechanisms related to inflammation and oxidative stress (Aleksandrova et al., 2021). In addition, legumes and vegetables, which are consumed more in the group of healthy patterns, are rich in antioxidants, as well as folate and B vitamins, which can reduce inflammatory reactions and oxidative stress and related with reducing aggression rates (Kamphuis et al., 2008; Wang et al., 2021). Also, adherence to the Western dietary pattern is related to the reduction of brain‐derived neurotrophic factor level, which is the protective factor of brain neurons against oxidative stress (Molteni et al., 2002; Shirayama et al., 2002).

Since examining the relationship between dietary patterns and aggression among female adolescents in Iran has occurred for the first time, there were several limitations which should be considered. Firstly, similar to other cross‐sectional studies, the exact causal link is not ascertained. Secondly, similar to other observational studies, this study has some confounders that could not be measured or controlled. The final limitation is inherent in the title of the research, namely that only females have been examined. On the other hand, examining a wide range of potential confounders together with the high quality with which data were collected and controlled, represent the strength of the findings of the current paper.

## 5 CONCLUSION

In conclusion, the present study indicates that a high adherence to a Western dietary pattern, characterized by high energy, red meat, processed meat, low and high fat dairy, soft drinks, snack and sweet and desserts but lower intake of legumes, spices, hydrogenated fat, and sugar was associated with increased risk of aggression in female adolescents. On the other hand, there is a need for food and nutrition policymakers to modify dietary patterns in adolescents towards healthy patterns to reduce possible psychological disorders. Further studies, especially longitudinal intervention studies using a larger sample sizes is needed to examine the association between adherence to major dietary patterns and aggression presence among children and adolescents.

## AUTHOR CONTRIBUTIONS

All authors made substantial contributions to conception and design, acquisition of data, or analysis and interpretation of data; took part in drafting the article or revising it. The study was conceived by Majid Ghayour‐Mobarhan, Gordon A. Ferns, and Naseh Pahlavani. The instruments were developed by Mahsa Malekahmadi, Sayyed Saeid Khayyatzadeh, Shadia Hamoud Alshahrani, Javad Heshmati, Nikzad Oraee, Majid Ghayour‐Mobarhan, Sayyed Saeid Khayyatzadeh, and Naseh Pahlavani. Data were collected by Sayyed Saeid Khayyatzadeh, Safieh Firouzi, and Mahsa Malekahmadi, with data analysis and interpretation by Shadia Hamoud Alshahrani, Mahsa Malekahmadi, and Naseh Pahlavani wrote the first draft of the manuscript. All authors read and approved the final version of the manuscript.

## CONFLICT OF INTEREST

The authors have no conflict of interest to disclose.

### PEER REVIEW

The peer review history for this article is available at https://publons.com/publon/10.1002/brb3.2782

## Supporting information


**Supplementary Table 1**. Food grouping used in the dietary patterns

**Supplementary Table 2**. Food loading matrix for major dietary patterns*

## Data Availability

The datasets used and/or analyzed during the current study are available from the corresponding authors on reasonable request.
