# Supplementary material for: The relationship between dietary patterns and aggressive behavior in adolescent girls: A cross‐sectional study
Source: Brain Behav. 2022 Oct 28;12(12):e2782. doi: 10.1002/brb3.2782 (PMC9759149; doi:10.1002/brb3.2782)
Supplement: Supplementary file 1 — Supplementary Table 1. Food grouping used in the dietary patterns [file BRB3-12-e2782-s002.docx]

| **Supplementary Table 1.** Food grouping used in the dietary patterns | |
| --- | --- |
| **Food groups** | **Food items** |
| Refined grains | White breads (lavash, baguettes), rice, Macaroni, noodles |
| Whole grains | Dark breads (Iranian), corn, Barley, bulgur |
| Potatoes | Potatoes |
| Snacks | French fries, chips, crackers |
| Legumes | Beans, peas, lentils, soy, mung, split peas |
| Other vegetables | broad beans, Cucumber, mixed vegetables, eggplant, celery, green peas, green beans, Sweet pepper, turnip, squash, mushrooms, carrots, onions |
| Red meats | Beef, hamburger, lamb, minced meat |
| Poultry | Chicken |
| Fish | Canned tuna fish, other fish |
| Organ meats | Heart, liver and kidney, intestine and viscera |
| Processed meats | Sausages |
| Eggs | Eggs |
| pizza | Pizza |
| Low fat dairy products | Skim or low-fat milk, low-fat yogurt |
| High fat dairy products | High-fat milk, whole milk, chocolate milk, cream, high-fat yogurt, cream yogurt, cream cheese, other cheeses, ice cream |
| Yoghurt drink | Doogh |
| Butter | Butter |
| Margarine | Margarine |
| Cruciferous vegetables | Cabbage, cauliflower, Brussels sprouts, Kale |
| Tomatoes | Tomatoes, red sauce |
| Green leafy vegetables | Spinach, lettuce |
| Garlic | Garlic |
| Fruits | Orange, tangerine, lemon, lime, grapefruit, banana, apple, pear, strawberry and other berries, peach, cherries, fig, melon, watermelon and Persian melon, cantaloupe, raisins or grapes, kiwi, apricots, nectarine, mulberry, plums, persimmons, pomegranates, date |
| Dried fruits | Raisins, dried berries, other dried fruits |
| Fruit juice | Lemon juice, All types of juice |
| industrial Juice and fruit compote | industrial Juice, fruit compote |
| Olives | Olives, olive oils |
| Hydrogenated fats | hydrogenated vegetable oils, animal oils |
| Vegetables oil | Vegetable oils (except for olive oil) |
| Mayonnaise | Mayonnaise |
| Nuts | Walnut, all types of nuts |
| Sugars | Sugar, candy |
| Soft drinks | Soft drinks |
| Sweets and desserts | Jam, Iranian confectioneries (gaz, sohan), chocolates, biscuits, Cakes, confections |
| Honey | Honey |
| Tea | Tea |
| Coffee | Coffee |
| Salt | Salt |
| Pickle | Pickle |
| Spices | Spices, green pepper |
