# Supplementary material for: The relationship between dietary patterns and aggressive behavior in adolescent girls: A cross‐sectional study
Source: Brain Behav. 2022 Oct 28;12(12):e2782. doi: 10.1002/brb3.2782 (PMC9759149; doi:10.1002/brb3.2782)
Supplement: Supplementary file 2 — Supplementary Table 2. Food loading matrix for major dietary patterns* [file BRB3-12-e2782-s001.docx]

| **Supplementary Table 2.** Food loading matrix for major dietary patterns* | | | |
| --- | --- | --- | --- |
|  | Dietary patterns | | |
| Food groups | First pattern | Second pattern | Third pattern |
| Refined grains | - | - | 0.22 |
| Whole grains | - | - | - |
| Potatoes | - | 0.21 | - |
| Snacks | - | 0.23 | 0.50 |
| Legumes | 0.25 | - | - |
| Other vegetables | 0.71 | - | - |
| Red meats | - | - | 0.35 |
| Poultry | - | - | 0.36 |
| Fish | 0.23 | - | 0.31 |
| Organ meats | - | - | 0.20 |
| Eggs | 0.32 | - | - |
| Pizza | - | - | 0.31 |
| Yoghurt | 0.40 | - | - |
| Butter | - | - | - |
| Margarine | - | - | - |
| Cruciferous vegetables | 0.44 | - | - |
| Tomatoes | 0.62 | - | - |
| Green leafy vegetables | 0.61 | - | - |
| garlic | 0.33 | - | - |
| Fruits | 0.32 | - | - |
| Dried fruits | - | - | - |
| Fruit juices | - | - | 0.30 |
| Industrial juice and compote | - | - | 0.48 |
| Olives | 0.25 | - | - |
| Hydrogenated fats | - | 0.37 | - |
| Vegetables oil | - | 0.26 | - |
| Mayonnaise | 0.23 | - | 0.26 |
| Nuts | - | - | 0.28 |
| Sugars | - | 0.63 | - |
| Soft drinks | - | 0.28 | 0.45 |
| Sweets and desserts | - | 0.25 | 0.43 |
| Honey | - | - | - |
| Tea | - | 0.65 | - |
| Coffee | - | - | 0.26 |
| Low fat dairy products | 0.39 | - | - |
| High fat dairy products | 0.35 | - | - |
| Salt | - | 0.62 | - |
| Pickle | - | - | 0.22 |
| Spices | - | 0.65 | - |
| Percent of variance explained | 8.69 | 5.59 | 4.1 |
| *Values less than 0.20 are not reported. | | | |
